# Simultaneous application of oral and intravaginal probiotics for *Helicobacter pylori* and its antibiotic-therapy-induced vaginal dysbacteriosis

**DOI:** 10.1038/s41522-024-00521-9

**Published:** 2024-06-20

**Authors:** Yufan Wang, Zhenyu Zhang, Qi Chen, Tingtao Chen

**Affiliations:** 1 Department of Obstetrics and Gynecology, The Second Affiliated Hospital, Jiangxi Medical College, Nanchang University, Nanchang, Jiangxi 330006 China; 2 Queen Mary School, Jiangxi Medical College, Nanchang University, Nanchang, 330031 China; 3 National Engineering Research Centre for Bioengineering Drugs and Technologies, Institute of Translational Medicine, Jiangxi Medical College, Nanchang University, Nanchang, 330031 China; 4 Department of Gastroenterology, Nanjing First Hospital, Nanjing Medical University, Nanjing, China; 5 School of Pharmacy, Jiangxi Medical College, Nanchang University, Nanchang, 330031 China

**Keywords:** Applied microbiology, Cellular microbiology, Clinical microbiology, Clinical microbiology, Microbiota

## Abstract

*Helicobacter pylori* is a prevalent bacterial pathogen globally, implicated in various gastrointestinal disorders. Current recommended antibiotic therapies for *H. pylori* infection have been proven to be therapeutically insufficient, with low eradication rates and high recurrence rates. Emerging evidence suggests that antibiotic therapy for *H. pylori* can lead to gastrointestinal and subsequent vaginal dysbiosis, posing challenges for conventional antibiotic approaches. Thus, this article proposes a novel probiotic therapy involving simultaneous oral and intra-vaginal probiotic administration alongside antibiotics for *H. pylori* treatment, aiming to enhance eradication rates and mitigate dysbiosis. We begin by providing an overview of gastrointestinal and vaginal microbiota and their interconnectedness through the vagina-gut axis. We then review the efficacy of current antibiotic regimens for *H. pylori* and discuss how antibiotic treatment impacts the vaginal microenvironment. To explore the feasibility of this approach, we evaluate the effectiveness of oral and intra-vaginal probiotics in restoring normal microbiota in the gastrointestinal and vaginal tracts, respectively. Additionally, we analyze the direct mechanisms by which oral and intra-vaginal probiotics act on their respective tracts and discuss potential cross-tract mechanisms. Considering the potential synergistic therapeutic effects of probiotics in both the gastrointestinal and vaginal tracts, dual-channel probiotic therapy holds promise as a more effective approach for *H. pylori* eradication and dysbiosis mitigation, presenting a novel concept in the collaborative treatment of gastrointestinal and genital disorders.

## Introduction

*Helicobacter pylori*, a gram-negative pathogenic bacterium, colonizes the gastrointestinal (GI) tract and is classified as a class I carcinogen by the International Agency for Research on Cancer (IARC), representing a major contributor to gastric cancer^1^. *H. pylori* infection is widespread in human population and its morbidity reaches 20% to 30% in developed areas, while in economically underdeveloped countries, the prevalence can be higher than 50%^2^. Studies have consistently shown that *H. pylori* can initiate chronic active gastritis in nearly all infected individuals, which may progress to peptic ulceration or gastric fibrosis^3^. Moreover, persistent infection significantly elevates the risk of developing precancerous lesions such as atrophic gastritis by approximately nine-fold, along with an eight-fold increase in the risk of actual carcinogenesis^4^.

Combinations of multiple antibiotic agents (clarithromycin, amoxicillin, and metronidazole) and proton pump inhibitors (PPIs) are widely used in the current regimen for *H. pylori* infection as first-line treatment^5^. The most commonly employed eradication therapies are triple or quadruple antibiotic regimens, which have demonstrated favorable clinical outcomes with an average eradication rate of 80% to 87%^6^. However, the overuse of antibiotics has led to several issues, including reduced efficacy of antibiotic treatment due to the escalation of drug resistance and a high recurrence rate^7^. Systemic antibiotic therapy can also be correlated with various side effects, such as allergic reactions, gastrointestinal symptoms, and gastrointestinal dysbiosis^8^. Additionally, the indiscriminate antimicrobial effect can further negatively impact the beneficial microbiota residing in other anatomical sites, leading to overall dysbiosis^9^.

A normal vaginal bacterial microbiota is typically dominated by *Lactobacillus* species, which creates a relatively low-pH biotic habitat containing lactic acid, bacteriocins, and other antibacterial molecules, playing an instrumental role in female urogenital health^10^. Oral antibiotics used in triple or quadruple therapies have broad spectrum of activity, and besides disturbing gastrointestinal microbiota^11^, they may also affect microbiomes in other parts of the body. Clinical practice has now confirmed that the regular application of antibiotics to treat *H. pylori* infection could lead to vaginal dysbiosis^12^. On the other hand, the unbalanced microecological environment of the human vagina, disturbed by antibiotics, can further cause a large amount of opportunistic pathogens such as *Candida albicans* to colonize and multiply, thus causing various vaginal inflammatory diseases and severely endangering female vaginal health^13^.

This review first provides an overview of gastrointestinal and vaginal microbiota, highlighting their close interconnection *via* the vagina-gut axis. Moreover, the influences and underlying mechanisms by which *H. pylori* eradication therapy endangers both gastric and vaginal microbiota are discussed, along with the limitations of current antibiotic therapy for vaginal dysbiosis. Recognizing the inadequacies of current therapeutic methods for *H. pylori* infection and dysbiosis, and considering the potential synergistic effect of oral and intra-vaginal administration of probiotics, we explore the prospects for the simultaneous dual-channel application of probiotic therapy in treating both *H. pylori* infection and alleviating the perturbation of antibiotics on the body microbiota. We then evaluate the therapeutic efficacy of current oral and intra-vaginal probiotic supplements in regulating body dysbiosis in both gastrointestinal and vaginal tracts. Finally, we discuss the potential difficulties and drawbacks of dual-channel probiotic therapy.

## Gastrointestinal microbiota and vaginal microbiota

### Overview of gastrointestinal and vaginal microbiome

The human gastrointestinal microbiome is a vast and dynamic ecosystem that plays a fundamental role in human health and well-being. Factors such as diet, age, exposure to microbes, and antibiotic application have all been linked to the initiation and preservation of microbial diversity within the gut^14^. With microbial cells outnumbering somatic cells by at least tenfold, the gut microbiome harbors a staggering diversity of microorganisms, collectively contributing far more genes than the human genome itself^15^. This intricate community of microbes influences various aspects of host physiology, immunity, and systemic nourishment, orchestrating a delicate balance known as homeostasis. Among the predominant taxa, Bacteroidetes, Firmicutes (including the genus *Lactobacillus*), Actinobacteria (including the genus *Bifidobacterium*), and Proteobacteria stand out^16^. While Fusobacteria, Saccharibacteria, Spirochaetes, and Synergistetes exhibited relatively lower abundance^16^. Zooming into the genus level, the most prevalent microbiota in the healthy human gut appears to be *Lactobacillus* and *Bifidobacterium*, owing to the vaginal microbiota during infant delivery and microbial species harboring in breast milk^17^. Within the *Lactobacillus* spp., *L. gasseri*, *L. casei*, and *L. rhamnosus* are dominant, while within the *Bifidobacterium* genus, *B. longum*, *B. bifidum*, and *B. adolescentis* are dominant in the gut microenvironment^18^. These microbial inhabitants interact with each other and with the host in a highly coordinated manner, shaping the gut environment and exerting profound effects on host health.

Similar to the gut microbiota, the initial colonization of the vaginal microbiota begins at birth, primarily comprising maternal vaginal and fecal microbiota^19^. Vaginal microbiota accounts for approximately 9% of the total microbiota of the human body^20^. Generally, the healthy female vagina harbors a diverse array of microorganisms, including *Candida albus*, *Gardnerella*, *Escherichia coli*, *Enterococcus*, *Streptococcus*, and *Staphylococcus* and other opportunistic bacteria, and also can be isolated with probiotics like *Bifidobacterium* and *Lactobacillus*^21^. The populations of these vaginal microbiomes are typically in dynamic equilibrium in healthy women of reproductive age^22^. The predominant bacteria in healthy adult vagina consist of *Lactobacillus* species (mainly *L. iners*, *L. crispatus*, and *L. gasseri*), with other microbiota present at lower abundance, such as *Peptostreptococcus* spp., *Corynebacterium*, *Bacteroides* spp., and Enterobacteriaceae^23,24^. *Lactobacillus* species produce lactic acid, which helps maintain an acidic pH in the vagina, inhibiting the growth of harmful bacteria and yeast. Additionally, the vaginal microbiome contributes to the production of antimicrobial peptides and the modulation of local immune responses^25^. These microbiotas constitute a crucial part of the microenvironment in the vagina, and the balance they establish is vital for immunity and providing shelter to their host.

### Association between gastrointestinal and vaginal microbiome

The gastrointestinal and vaginal microbiomes are two distinct microbial ecosystems within the human body, each with its own unique composition and functions. However, emerging research suggests that there may be interconnectedness between these microbiomes. The concept of the “gut-vaginal axis” proposes a bidirectional communication pathway between the gut and vaginal microbiomes^26^. It suggests that changes in the gut microbiome composition can influence the vaginal microbiome and vice versa.

Both the vaginal and gastrointestinal tracts serve as major colonization sites for numerous species of bacteria within the body. Their initial colonization typically originates from maternal vaginal and fecal microbial species^19^. Similar to the microbiota present in the mother’s vagina, the majority of microbes found in the meconium of infants delivered vaginally are *Lactobacillus* and *Prevotella*, revealing that maternal vaginal microbiota may serve as one of the sources of gut microbiota in infants^27^. This may also account for the strong similarities observed in their taxa composition in adults. In the vaginal tract, facultative anaerobic *Lactobacillus* is the dominant bacterial group, while in the GI tract, both facultative anaerobic *Lactobacillus* and strict anaerobic bacterium *Bifidobacterium* are dominant^28^. These microorganisms play similar roles in maintaining human health within their respective microecosystems and can cause gastrointestinal or vaginal disorders when the normal microbiota is disrupted^29^.

**Fig. 1:**
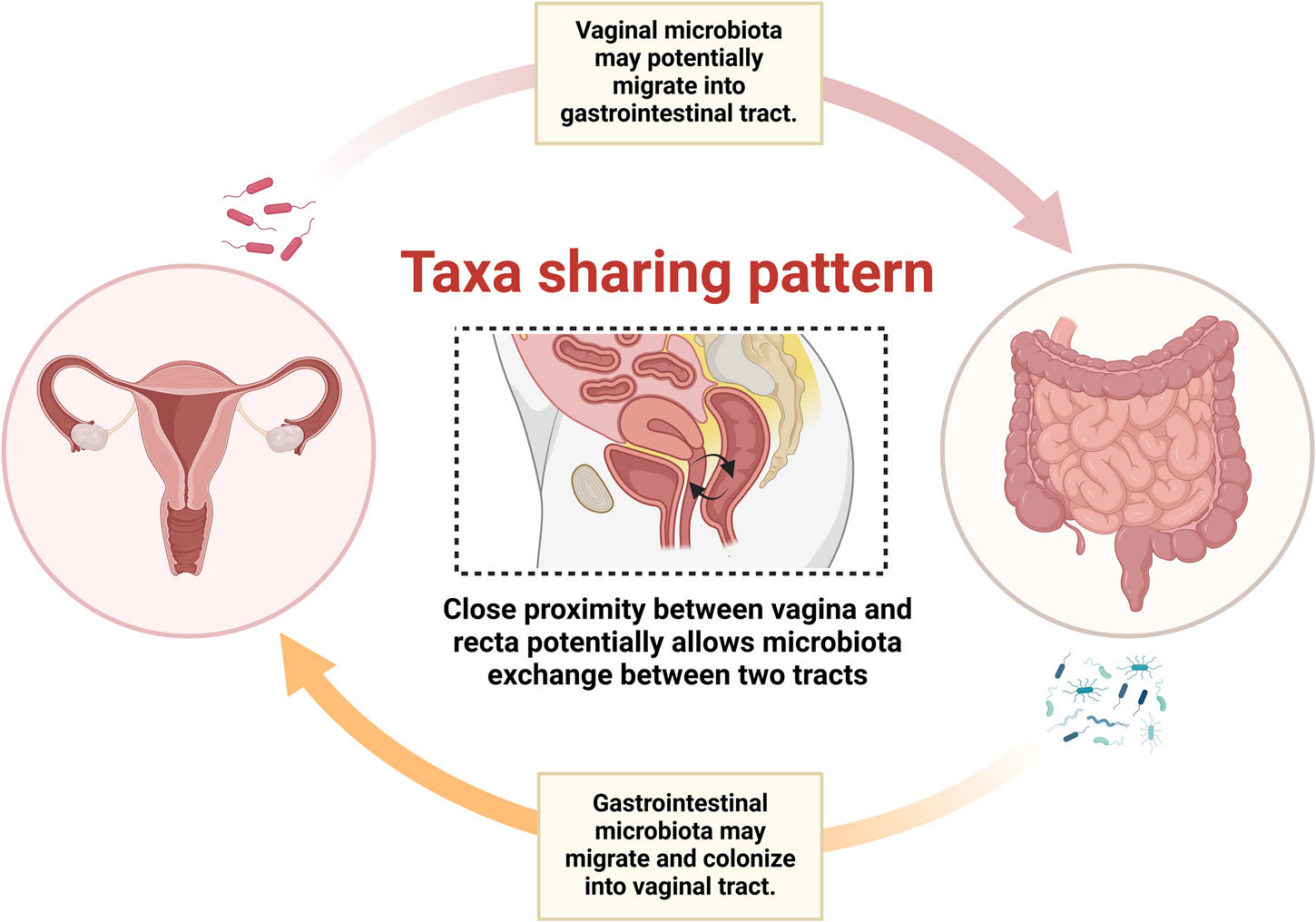
Schematic of taxa sharing pattern between gastrointestinal tract and vaginal tract. Direct microbial translocation between the rectum and vagina could be a potential mechanism through which oral or intra-vaginal probiotic administration affects the opposite tract.

Furthermore, the close anatomical distance between the rectum and vagina may facilitate the trafficking of microorganisms across the gut and vagina (Fig. 1). A previous study suggested that certain H_2_O_2_-producing *Lactobacillus* strains are prevalent in both the vaginal and rectum tract, contributing to the normal maintenance of vaginal microbiota^30^. Moreover, another study indicated that out of the 66 bacterial species identified in the vagina and rectum, 44% were found in both tracts and the genotypes of 68% these species were identical. Furthermore, utilizing quantitative PCR, Aila and colleagues suggested a significant correlation between the quantities of rectal and vaginal *L. crispatus*, *L. jensenii*, *L. gasseri*, and *L. iners*, implying a close association between rectal and gut microbiota^31^. These pieces of evidence support the notion that the rectum could be responsible for the storage and reservation of vaginal microorganisms, and possibly vice versa.

Despite direct microbial migration, indirect associations between vaginal and gut microbiota could also be implicated. Metabolites produced by gut microbial species, such as short-chain fatty acids (SCFAs), could play a role in the vagina-gut axis, as they can be transferred to other anatomical sites through the general circulation^28^. The elevated SCFAs in the vagina may indicate vaginal dysbiosis and provoke a proinflammatory response^32^, suggesting that the circulation of SCFAs from the gut to the vagina may disturb the vaginal microenvironment. Additionally, sex hormones such as estrogen can also play a role in vagina-gut axis. Gut microbiota, including *Bifidobacterium*, *Clostridium*, and *Lactobacillus*, are involved in the metabolism of estrogen, contributing to the deconjugation of estrogens^33^. Deconjugated estrogen can facilitate the production of glycogen within vaginal tract *via* systemic circulation, further stimulating the proliferation of vaginal *Lactobacillus*^34^. Therefore, the abundance of gut bacteria associated with estrogen metabolization could correlated with the abundance of vaginal *Lactobacillus* species.

Several studies could also support the notion that the alternations in gut microbiota could mirror in the vaginal microbiota. Based on an animal trial^35^, approximately half of the taxa (48%) exhibited enrichment in vagina post oral antibiotic treatment, while a distinct reduction in the gut (*Erysipelothrix*, *Roseburia*, *Anaerotruncus*, and *Akkermansia*). While for Actinobacteria and Proteobacteria, it showed an opposite result that they enrich in vagina but deplete in gastrointestinal tract after antibiotic treatment. Conversely, in a clinical trial, subjects with vaginal candidiasis demonstrated not only the disturbance of vaginal microbial profile but also in gut microbial community, resulting in a depletion in gut microbial diversity^36^. This suggests that alternations in vaginal microbiota can also in turn affect the gastrointestinal microbiota. Collectively, the physiological eubiosis between gastrointestinal and vaginal microbiota could be tightly correlated through the vagina-gut axis, whereby changes in one tract could affect the other.

## Impact of *Helicobacter pylori* infection and antimicrobial therapy on microbial dysbiosis

### *Helicobacter pylori* infection and its current eradication therapy

*H. pylori* was initially identified by Marshall and Warren in 1984 through observation of antral mucosa tissue sections from a patient with chronic gastritis^37^. Upon noticing the presence of inflammation in the adjacent gastric mucosa, Warren hypothesized that *H. pylori* might be closely associated with the incidence of gastritis. In the majority of cases, initial complications resulting from *H. pylori* infection typically lead to mild pangastritis, which does not significantly affect gastric physiology or lead to severe diseases^38^. However, *H. pylori* can establish persistent infection in the acidic environment of the gastric mucosa due to its unique residing characteristics, such as paralogous outer membrane proteins (OMPs)^39^ and the combination of urase and urea channel (UreI)^40^. If left untreated, persistent infection can progress to antral predominant gastritis^41^, chronic non-atrophic gastritis^42^, and even atrophic gastritis^43^, which is considered a major precancerous lesion for gastric cancer. Further progression depends on the virulence of pathogenic strains, such as the *cag*-pathogenicity island (*cag*-PAI)^44^. Expression of *Cag*-PAI genes can lead to the deterioration of normal gastric epithelium and ultimately result in intestinal metaplasia. These developments may gradually progress into noninvasive neoplasia, high-grade dysplasia, and eventually invasive malignant gastric carcinoma, which can be extremely harmful and even fatal^45^.

In 2007, the American College of Gastroenterology Guideline proposed standard triple therapy as the first-line regimen for *H. pylori* infection, consisting of a proton pump inhibitor (PPI)^46^ and two other antibacterial agents (clarithromycin and amoxicillin) for a 2-weeks course^6^. Later, sequential therapy is commonly recommended as an alternative to standard triple therapy due to high drug resistance. This regimen involves 5 days of PPI and amoxicillin, followed by an additional 5 days of PPI along with two different antibiotics (typically clarithromycin and metronidazole)^47^. For the latest recommendations, the Toronto Consensus guidelines^48^ and Maastricht V/Florence Consensus Report^49^ proposed bismuth-based therapy as the newest first-line treatment, which involves adding bismuth to triple or quadruple therapy. Generally, current therapeutic methods tend to favor therapies with longer medication courses, higher antibiotic doses, and additional new adjuvant antibiotics to address the rising antibiotic resistance.

The drug resistance of *H. pylori* to key antibiotics such as clarithromycin, metronidazole, levofloxacin, and amoxicillin in conventional standard treatment has continued to increase over the past twenty years, significantly reducing the eradication rate^50,51^. In addition to antibiotic drug resistance, high rates of recrudescence^52^, severe complications^53^, and local dysbiosis induced by antibiotic administration further pose significant challenges for *H. pylori* eradication.

### *Helicobacter pylori* infection and eradication affect gastrointestinal microbiomes

It has been reported that *H. pylori* infection can alter the diversity of the intestinal microbiota^54^. This may be due to changes in the pH of the gastrointestinal caused by *H. pylori* infection, leading to damage and invasion of the gastrointestinal mucosa. Further, compromised gastric mucosa may increase the adhesion and migration of immune cells, ultimately resulting in the inability of the original gastrointestinal microbiota to survive^55^. From one clinical trial, the gut microbial diversity was significantly reduced in individuals infected with *H. pylori* compared to healthy individuals, with significantly decreased abundance of Actinobacteria, Bacteroidetes, Firmicutes, Fusobacteria, Gemmatimonadetes, and Verrucomicrobia. Additionally, eight genera were significantly more abundant in healthy individuals compared to those with *H. pylori* infection, including *Achromobacter*, *Devosia*, *Halomonas*, *Mycobacterium*, *Pseudomonas*, *Serratia*, *Sphingopyxis*, and *Stenotrophomonas*^56^. Moreover, another study reported a reversal in gastric microbial abundance at the phylum level. Reduced bacterial diversity was observed in *H. pylori* subjects, with Proteobacteria, Firmicutes, Bacteroidetes, and Actinobacteria being the most abundant phyla, whereas in normal subjects, the most abundant phyla were Bacteroidetes, Firmicutes, Actinobacteria, and Proteobacteria^57^. These alterations in the gastrointestinal microbiota, driven by *H. pylori* infection, may lead to the progression of gut dysbiosis.

Another implicated issue of gut dysbiosis is provoked by the indiscriminate antimicrobial effects of *H. pylori* eradication therapy^58^. Both antibiotics and PPIs used for *H. pylori* eradication may significantly impact the gut microbiota due to their antimicrobial effects and their ability to reduce gastric acidity. The administration of antibiotics on gut microbiota may lead to the reduction of gut microbial diversity, decreased abundance of certain taxa, and increased risk of gut infection^59^. A meta-analysis suggested that while *H. pylori* eradication therapy successfully eliminates *H. pylori*-related taxa, the restoration of gut microbiota to a normal microecological status remains controversial^60^. One clinical trial assessed the long-term impact of gut microbiota following treatment with three different *H. pylori* eradication therapies (standard triple therapy, concomitant therapy, and bismuth quadruple therapy). The alpha diversity and beta diversity of gut microbiota were significantly altered in all three regimens 2 weeks post-treatment. Alpha diversity and beta diversity were restored in the standard triple therapy group at week 8 and 1 year post-treatment, while failed to restore in the concomitant therapy and bismuth quadruple therapy groups at week 8 and even 1 year after eradication^61^. Moreover, another study revealed that notable alterations in the taxonomic composition of gut microbiota persisted even two months after administering a triple therapy based on vonoprazan, albeit with a restoration of microbial diversity^62^. Future investigations are required to develop an eradication regimen with sufficient efficacy against *H. pylori* while minimizing disruption to the gut microbiota.

## *Helicobacter pylori* eradication therapy and vaginal dysbacteriosis

### Evidence of *Helicobacter pylori* antimicrobial therapy induced vaginal dysbiosis

In addition to gastrointestinal microbiomes, the antimicrobial effects of antibiotics could also affect the microbial communities in skin^63^, respiratory^64^, and vagina^65^. Antibiotics used to treat *H. pylori* infections typically aim to eradicate bacterial colonization but without specifically targeting particular microbiomes. Due to their broad antimicrobial spectrum, these antibiotics may have off-target effects, resulting in concentrations exceeding what is necessary for eliminating pathogenic *H. pylori*. Consequently, they could disrupt and imbalance the normal body microbiota, reducing colonization resistance for an extended period following administration^66^.

A clinical study by Kravtsov et al.^12^ aimed to investigate the possibility of an increased risk of candidiasis in the female genital tract after *H. pylori* eradication therapy. They reported that following a 2-week quadruple bismuth therapy (consisting of rabeprazole, amoxicillin, tetracycline, and bismuthate tripotassium dicitrate), elements of *Candida* fungus were found in smears taken from the cervix uteri and lateral vaginal vault in all patients. Approximately 22% of patients administered anti-helicobacter therapy were diagnosed with *Candida* vulvovaginitis, indicating an increased incidence of vaginal dysbiosis after *H. pylori* eradication. Further investigation revealed significantly elevated levels of cytokines IL-8 and TNF-α in vaginal secretions from patients treated with anti-helicobacter therapy compared to those without antibiotic administration^67^. This suggests that *H. pylori* eradication treatment may strongly disrupt the immune status of the female vaginal tract. In a Chinese clinical comparative study involving 15 female patients with *H. pylori* infection, after undergoing standard triple therapy (rabeprazole, amoxicillin, and levofloxacin), 7 participants experienced an imbalance in vaginal microecology, and 5 exhibited fungal overgrowth^68^. Similarly, a different triple eradication therapy comprising omeprazole, amoxicillin, and metronidazole revealed a decrease in the normal rates of vaginal cleanliness, pH balance, and the abundance of *Lactobacillus* species^69^. Additionally, levels of vaginal secretory immunoglobulin A (SIgA) were found to be elevated following treatment. These findings indicate a significant correlation between standard *H. pylori* eradication therapies and alternations in the vaginal microecology of female patients, suggesting that antibiotic treatments for *H. pylori* could disrupt the delicate equilibrium of the vaginal microbiome.

However, the clinical evidence supporting the notion that *H. pylori* eradication therapy can lead to dysbiosis of the vaginal microbiota is still lacking. This may be attributed to the general lack of focus on vaginal outcomes following oral therapy, as well as the insufficient research on the vaginal-gut microbiota axis. However, in clinical practice, a prominent proportion of patients with gynecological disorders experiencing vaginal dysbiosis are observed after their *H. pylori* eradication therapy. Additionally, there are numerous reports of oral antibiotics causing disruptions in vaginal microbiota^70^. Therefore, it is reasonable to expect that *H. pylori* eradication therapy may increase the risk of vaginal dysbiosis in patients.

Next-generation whole-genome shotgun sequencing and targeted sequencing have revealed that antibiotic exposure can lead to a decrease in vaginal microbial diversity, total biomass, and functional diversity^71^. Additionally, Pirotta et al. found that the rate of vaginal *Candida* species infection significantly increased from 21% to 37% after treatment with amoxicillin^13^. Similarly, in a study by Kurowski et al., 12 female patients treated with clarithromycin showed a decrease in *Lactobacillus* culture from 33% to 0 after treatment, while the rate of vaginal *Candida* infection increased from 17% to 33% post-antibiotic treatment^72^. Moreover, another study reported a significant increase in the colonization rate (83%) of vaginal *Staphylococcus* species in pregnant women after oral antibiotic administration compared to women without antibiotic treatment (76%)^73^. Furthermore, an animal trial demonstrated that the vaginal microbial profile in mice was significantly altered after oral antibiotic treatment, with depleted abundance of Actinobacteria and Proteobacteria, and enriched abundance of Tenericutes and Bacteroidetes^35^. Therefore, it can be inferred that antibiotic applications in *H. pylori* infection could disrupt normal vaginal microecology, leading to a decrease in beneficial microbiota and an increase in opportunistic bacteria, which may contribute to various gynecological disorders.

The mechanism by which oral antibiotics induce vaginal dysbiosis is still unclear, but it may account for the general circulation or the shared microbiota between gut and vaginal taxa as discussed as vagina-gut axis^26^. One potential hypothesis for this may be that PPIs alter the pH of the gastrointestinal tract, allowing certain bacteria to proliferate extensively in the intestines, leading to dysbiosis, such as *Candida* species^12^. Additionally, the use of antibiotics can affect the microbial composition on the surface of the genital tract mucosa, thereby changing the immunity of the genital tract, allowing the overgrowth gastrointestinal bacteria to enter the vagina. In addition, orally administered antibiotics for *H. pylori* infection can be directly delivered into the intestinal lumen. After absorption and modifications in the liver, they either enter enterohepatic circulation and are excreted into feces or return into the blood for renal clearance and then enter the genitourinary tract. Both endings can be direct or indirect pathways by which board-spectrum antibiotics affect the vaginal microenvironment^74^. Antibiotics excreted through feces can directly impact the gastrointestinal tract, where they can disrupt the composition and balance of the gut microbiota. Disruption of the gut microbiota can lead to dysbiosis and the proliferation of opportunistic pathogens, which may then directly translocate to the genitourinary tract from anus^75,76^. Additionally, antibiotics cleared through renal excretion may initially enter the bloodstream before being filtered by the kidneys and subsequently excreted into the urine. However, some antibiotics may retain their antimicrobial effects when reaching the genitourinary tract. Upon entering genitourinary tract, these antibiotics may directly affect the local microbiota, including the vaginal microbiota, potentially leading to dysbiosis.

### Understanding vaginal dysbiosis and therapeutic challenges

Normal vaginal microbiota plays a crucial role in maintaining a healthy vaginal microenvironment^77^. The evidence mentioned above demonstrates that broad-spectrum antibiotics administered for *H. pylori* eradication could disrupt the vaginal microbiota, leading to vaginal dysbiosis^78^. Baeten and colleagues also suggested that recent antibiotic use was a risk factor for loss of vaginal *Lactobacillus*, which is correlated with the occurrence of dysbiosis^79^.

Bacterial vaginosis (BV) is a prevalent gynecological disorder worldwide, characterized by symptoms such as malodorous vaginal discharge and itching sensation around the vagina^80^. BV not only affects women’s self-esteem but also increases the risk of sexually transmitted infections (STIs)^81^. Furthermore, abnormal vaginal microbiota is also associated with an increased risk of preterm birth^82^, miscarriage^83^, and even infertility^84^. The currently recommended clinical regimen for treating BV is limited and commonly involves antibacterial agents, including metronidazole, nitroimidazole, tinidazole, or clindamycin^85^. First-line treatments typically consist of either a 7-day course of 500 mg oral metronidazole twice a day or a 5-day course with intra-vaginal metronidazole cream once daily^86^.

Though with relatively favorable cure rate, high recurrence poses a significant challenge for current antibiotic regimen. The self-formed biofilms and the development of antibiotic resistance among bacteria associated with BV, such as *Gardnerella vaginalis*, may play crucial roles in persistence and recurrence of the condition^70^. Within 6 to 12 months post-antibiotic therapy, 30% to 80% of patients experience recurrence^87^. For example, Rose et al. reported that 71% of patients had recurrent symptoms after completion of treatment with metronidazole^88^. Plummer and colleagues found that 17% of women experienced relapse 12 weeks post-metronidazole and clindamycin treatment^89^. Additionally, Aguin et al. reported that although only 1% experienced recurrence at the third month with high-dose therapy, 50% of patients still had recurrence 3 months after treatment^90^. The inability to restore the colonization of antimicrobial *Lactobacillus* species in the vaginal microenvironment following antibiotic therapy may serve as a critical reason contributing to the high recurrence rate. While antibiotic therapy decreases the abundance of *G. vaginalis* and other pathogens associated with BV, the microbiota following antibiotic treatment typically show dominance of *L. iners* rather than the species deemed more beneficial, such as *L. crispatus* and *L. jensenii*^91^. Therefore, probiotic therapy might be an alternative or adjunct method for conventional antibiotic therapy to achieve persistent cure and effectiveness for both bacterial vaginosis and *H. pylori* infection.

## Dual-channel probiotic therapy: a promising approach for addressing *Helicobacter pylori* infection and bacterial vaginosis simultaneously

Both *H. pylori* infection and bacterial vaginosis induced by prior *H. pylori* antibiotic therapy can lead to persistent and intractable symptoms in female patients. Moreover, the current antibiotic regimens for both conditions are often inadequate, resulting in high recurrence rates and various adverse effects^92^. Therefore, considering the interconnected nature of the microenvironments in the gastrointestinal and vaginal tracts, as well as the increasing recommendations for probiotic therapy in both *H. pylori* infection and bacterial vaginosis, dual-channel probiotic therapy could emerge as a promising approach to treating both diseases while simultaneously reducing the incidence of antibiotic-induced dysbacteriosis.

**Fig. 2:**
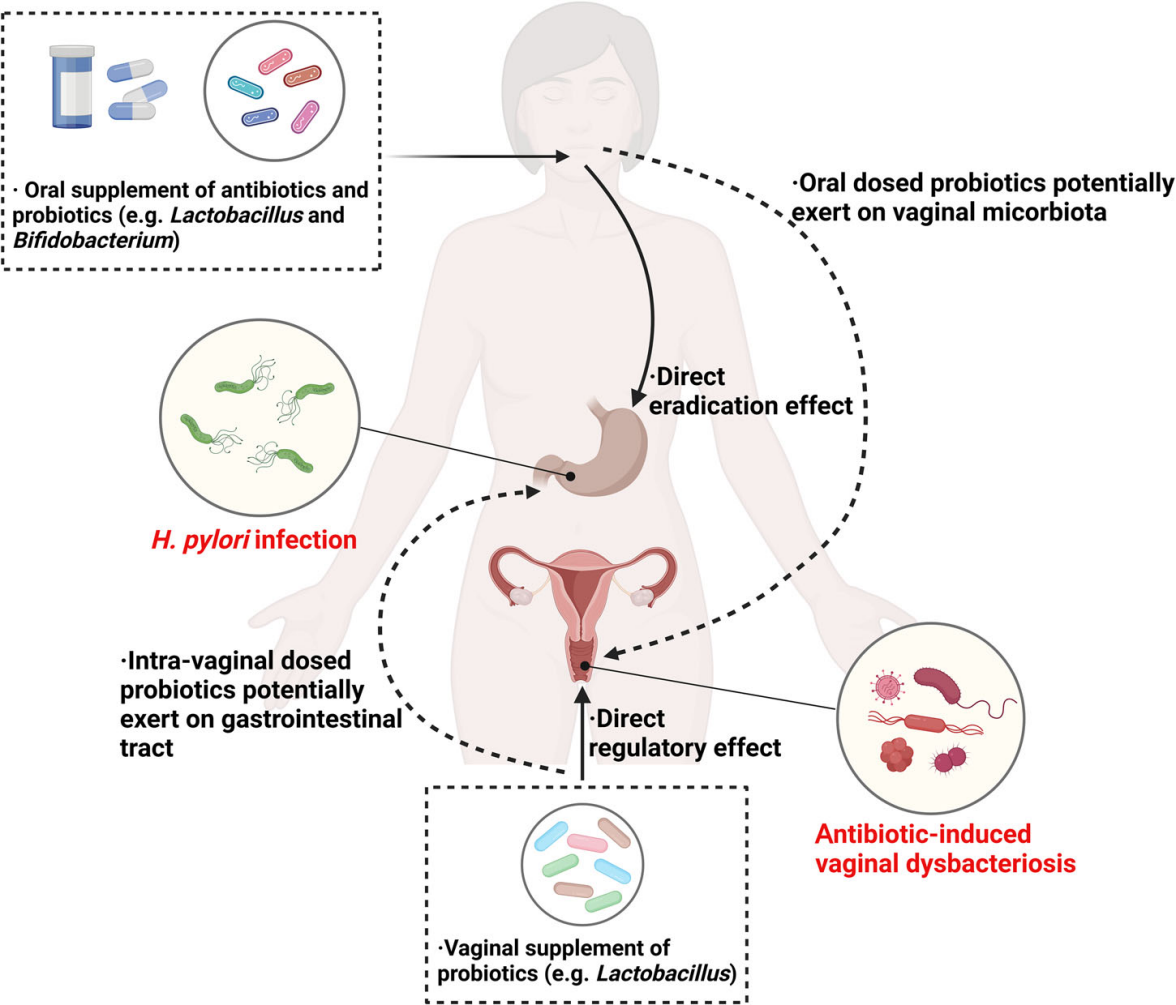
Schematic representation of dual-channel probiotic therapy. Oral administration of antibiotics and probiotics can directly regulate gastrointestinal dysbiosis and eradicate *H. pylori* infection, while also having an impact on vaginal dysbiosis. Concurrently, vaginal probiotics can mitigate the risk of antibiotic-induced vaginal dysbiosis and potentially modulate the gastrointestinal microbiota.

Dual-channel probiotic therapy involves the concurrent use of orally administered probiotics and antibiotics for *H. pylori* infection alongside intra-vaginally administered probiotic supplements (Fig. 2). By delivering probiotics through both channels, the efficacy of antibiotic eradication for *H. pylori* could be significantly enhanced, and the occurrence of antibiotic-related gastrointestinal and vaginal dysbacteriosis can be empirically reduced.

The concept of dual-channel probiotic therapy stems from the similarities between the gastrointestinal and vaginal tracts and their physiological interconnection. Both tracts serve as colonization sites for various bacterial species, with notable similarities in their taxonomic compositions. *Lactobacillus* species, predominant in the vaginal microbiota^93^, also play a significant role in the gastrointestinal tract^94^. The dominant *Lactobacillus* species in healthy vagina was considered originated from gut^95^, while the primary colonization of gut microbiota was originated from vertical transmission of microbiota from maternal vagina^96^. As discussed earlier, the vaginal microbiota and gastrointestinal microbiota are strongly correlated with each other through the vagina-gut axis. The anatomical proximity of the gut and vagina facilitates potential interactions between their microbiomes^97^. One species-level Spearman correlation coefficient analysis has revealed common BV-associated bacteria in both the rectal and vaginal tracts, suggesting a strong interconnection between their local microbiota^98^. Additionally, orally administered probiotic strains have been shown to colonize the vaginal tract^99,100^, indicating the possibility of recto-vaginal translocation for certain microorganisms. As observed in group B *Streptococcus* and *E. coli* infections^31^, similar mechanism of recto-vaginal translocation may apply for BV-associated microorganisms.

In addition to direct translocation of microbiota between two tracts, metabolites such as SCFAs^32^ and sex hormones^101^ may indirectly affect vaginal microbiota *via* the gut microbiota. Animal studies have demonstrated that vaginal microbiota can influence colonic levels of inflammatory markers and alter the gastrointestinal microbiota composition^102^. Furthermore, vaginal microbiota transplantation, a novel approach grounded on the resemblance between the vaginal and gastrointestinal tracts, and originating from fecal microbiota transplantation (FMT), has also been demonstrated to be efficacious in the treatment of BV^103^.

Moreover, probiotics in both tracts exert similar protective effects on mucosal epithelial cells through the production of bacteriocins, hydrogen peroxide, and organic acids^104^. They also compete with pathogens for nutrients and colonization sites, activate host immune defense systems, and regulate inflammatory signaling molecules to combat diseases^105,106^. Studies have also shown promising efficacy for *Lactobacillus* species in both vaginal and gastrointestinal dysbiosis (Table 1), supporting the feasibility of dual-channel probiotic therapy^107–109^. By integrating oral and intra-vaginal probiotic supplementation, this innovative approach may provide synergistic and high efficacy for *H. pylori* treatment while simultaneously reducing dysbacteriosis outbreaks in both the gastrointestinal and vaginal tracts.

**Table 1:** Performance of oral and intra-vaginal probiotics in regulating gastrointestinal and vaginal microbiota

Administration route	Experiment subjects	Intervention	Acting site	Outcomes	Refs.
Oral	Total 90 *H. pylori*-positive patients	All patients received triple therapy for 2 weeks before being administered either a probiotic or a placebo. Probiotic group received capsule (200 mg) of *L. reuteri* DSM 17648 strain supplement once daily.	Gastric lumen	Eradication rate: Probiotic 91.1% vs. Placebo 68.9%, *p* = 0.007. Adverse headache: Probiotic 0% vs. Placebo 15.6%, *p* = 0.012. Abdominal pain: Probiotic 0% vs. Placebo 13.3%, *p* = 0.026.	Ismail et al.^123^
Oral	Total 100 *H. pylori* infected patients	All patients received 7-or 14-day bismuth-containing quadruple therapy with either a probiotic or a placebo supplement. Probiotic group received tablet (37.5 mg) of *L. reuteri* DSM17938 and *L. reuteri* ATCC PTA6475 strain BID.	Gastric lumen	14-day eradication rate: Probiotic 96% vs. Placebo 88%. 14-day nausea and vomiting: Probiotic 6% vs. Placebo 26%, *p* = 0.002. 14-day abdominal discomfort: Probiotic 4% vs. Placebo 18%, *p* = 0.017.	Poonyam et al.^119^
Oral	Total 276 *H. pylori*-positive patients	All patients received *Bifidobacterium* Tetravaccine Tablets or placebo adjunct to 14-day bismuth-containing quadruple therapy. Probiotic tablets contain *B. infantis* CGMCC0460.1, *L. acidophilus* CGMCC0460.2, *Enterococcus faecalis* CGMCC0460.3, and *Bacillus cereus* CGMCC0460.4.	Gastric lumen	Gastrointestinal adverse events: Probiotic 23.6% vs. Placebo 37.7%, *p* = 0.016. Eradication rate: Probiotic 86.6% vs. Placebo 87.8%, *p* = 0.797. The relative abundance of *Bacteroides* was significantly reduced in the placebo group while restored in the probiotic group, accompanied by the enrichment of *Lactobacillus*, *Prevotella*, and *Bifidobacterium*.	He et al.^25^
Oral	Total 741 *H. pylori*-infected patients	All patients received 10-day non-bismuth containing quadruple therapy with either a probiotic or a placebo supplement. Probiotic group received combined strains of *L. acidophilus*, *Lactiplantibacillus plantarum*, *B. lactis*, and *Saccharomyces boulardii*.	Gastric lumen	Eradication rate: Probiotic 92.0% vs. Placebo 86.8%, *p* = 0.028. Adverse effects: Probiotic 17.0% vs. Placebo 50.7%, *p* < 0.00001.	Viazis et al.^162^
Oral	Total 450 *H. pylori* infected patients	All patients received 10-day non-bismuth containing quadruple therapy with either a probiotic or a placebo supplement. Probiotic group received supplement of *L. ruteri* (100 mg) twice daily.	Gastric lumen	Eradication rate: Probiotic 78.7% vs. Placebo 72.0%, *p* = 0.126. Heart burn: Probiotic 15.1% vs. Placebo 51.1%, *p* < 0.001. Abdominal pain: Probiotic 13.3% vs. Placebo 38.7%, *p* < 0.001. Loss of appetite: Probiotic 12.9% vs. Placebo 60.0%, *p* < 0.001.	Mohtasham et al.^163^
Intra-vaginal	Total 250 non-pregnant women diagnosed with bacterial vaginosis	All patients received 7-day standard metronidazole therapy BID with either a probiotic or a placebo supplement. Probiotic group received supplement of vaginal tablets containing *L. rhamnosus* BMX 54.	Vaginal tract	2-month restoration of vaginal microbiota: Probiotic 90.4% vs. Placebo 79.2%, *p* = 0.014. 6-month restoration of vaginal microbiota: Probiotic 74.6% vs. Placebo 25.4%, *p* < 0.0001. 9-month restoration of vaginal microbiota: Probiotic 79.7% vs. Placebo 20.3%, *p* < 0.001.	Recine et al.^164^
Intra-vaginal	5 women diagnosed with BV	All of 5 women applied with vaginal microbiota transplantation from healthy women.	Vaginal tract	4 of 5 achieved a long-term recovery from bacterial vaginosis with low Amsel criteria and alleviate from BV-related symptoms. One demonstrated an incomplete remission. All patients showed no adverse effects.	Lev-Sagie et al.^165^
Intra-vaginal	Total 90 women infected Trichomonas vaginalis with presence of BV	All patients first received 7-day metronidazole therapy BID with either a probiotic or a placebo supplement. Probiotic group received supplement of *L. rhamnosus* Lcr35. Both groups received probiotics for next 7 days.	Vaginal tract	Overall recover rate: Probiotic 88.6% vs. Placebo 42.9%. Day 8 presence of *T. vaginalis*: Probiotic 6.8% vs. Placebo 47.6%. Day 15 presence of *T. vaginalis*: Probiotic 11.4% vs. Placebo 57.1%. Day 8 presence of BV: Probiotic 9.1% vs. Placebo 38.1%. Day 15 presence of BV: Probiotic 9.5% vs. Placebo 63.6%.	Sgibnev et al.^108^
Intra-vaginal	Total 98 women diagnosed with at least two episodes of BV	All patients first received 7-day metronidazole therapy once a day. Patients then vaginally treated with either a probiotic or a placebo capsule for next 14 days. Probiotic group received supplement of *L. crispatus* IP 174178.	Vaginal tract	Recurrence rate: Probiotic 20.5% vs. Placebo 41.0%, *p* = 0.0497. Time to recurrence: Probiotic 3.75 ± 0.16 months vs. Placebo 2.93 ± 0.18 months, *p* = 0.0298.	Bohbot et al.^134^
Intra-vaginal	Total 117 women who had a BV with concomitant HPV infection	All patients first received standard therapy (metronidazole for 7 days or fluconazole for 2 consecutive days). Patients then vaginally treated with *L. rhamnosus* BMX 54 for either 3 months or 6 months.	Vaginal tract	Total HPV-clearance: Probiotic long-term group 31.2% vs. Probiotic short-term group 11.6%, *p* = 0.044.	Palma et al.^135^
Oral	BALB/c female mice and their offspring	Antibiotic group: Receive daily oral penicillin (31 mg/kg) for one week before the birth of offspring. Probiotic group: Same antibiotic treatment with daily *L. rhamnosus* JB-1 supplement (1×10^9^ CFU/d). Control group: Regular water and food. After birth, analyses the GI phyla of offspring after 6-weeks normal feeding.	Gastrointestinal tract	In antibiotic group, the GI phyla in offspring demonstrated a significant increment of relative abundance of Firmicutes and Lachnospiraceae while a decrement of Bacteroidetes and Prevotellaceae comparing with probiotic group and control group. The results indicated that with probiotic supplement, the prior maternal GI dysbiosis can be restored in their offspring.	Leclercq et al.^166^
Oral	4-week-old C57BL/6 J male mice	All mice first received cefixime gavage for 2 weeks and 16 s rDNA sequencing for GI microbiota. Probiotic group: Received probiotic cocktail treatment (*L. plantarum*, *L. casei* and *L. rhamnosus*) for next 4 weeks. Natural recovery group: Received normal food and water.	Gastrointestinal tract	After cefixime application, the general microorganism diversity is decreased. And the relative abundance of *Lactobacillus*, *Butyricicoccus* and *Parabacteroides* were reduced in GI environment, while increased of *Enterococcus*. The restoration speed is significant faster in probiotic treatment group compared with natural recovery group.	Shi et al.^167^
Oral	Total 36 women with bacterial vaginosis	All patients first received 7-day metronidazole therapy BID. Patients then received either placebo or yoghurt supplement (containing *L. crispatus* DSM 22566, *L. gasseri* DSM 22583, *L. jensenii* DSM 22567 and *L. rhamnosus* DSM 22560) twice daily for 4 weeks.	Vaginal tract	Amsel score: Probiotic 4.0 vs. Placebo 2.0, *p* = 0.038. Discharge and odor: Probiotic 0.0 vs. Placebo 1.0, *p* = 0.001. Nugent score: Probiotic 5.5 vs. Placebo 3.0, *p* = 0.158.	Laue et al.^168^
Oral	Total 36 asymptomatic womendiagnosed with vaginal dysbiosis	All patients received orally probiotics supplement (*L. acidophilus* CBT LA1, *L. rhamnosus* CBT LR5, and *L. reuteri* CBT LU4) for 6 weeks	Vaginal tract	Patients with high Nugent score demonstrated significantly improved vaginal dysbiosis with enriched microbial diversity and *Lactobacillus* spp. colonization in vagina.	Ansari et al.^138^
Oral	Total 89 women diagnosed with bacterial vaginosis and 93 women diagnosed with vulvovaginal candidiasis (VVC)	All patients were treated with oral or vaginal probiotic capsules, or placebo capsules for 3 months. BV patients received *L. crispatus* DSM32717 and *L. crispatus* DSM32720. VVC patients received *L. crispatus* DSM32720, *L. crispatus* DSM32716 and *L. crispatus* DSM32718.	Vaginal tract	VVC patients with orally administrated probiotics demonstrated significantly improved two main symptoms (discharge and itching/irritation).	Mändar et al.^139^
Oral	Total 93 women diagnosed with VVC	All patients were treated with *L. plantarum* P17630 or placebo for 30 days for 3 treatment cycle (15 days on, 15 days off).	Vaginal tract	Patients with orally administrated probiotics demonstrated significantly improved Lactobacillary grade (LBG) score at day 45 (*p* = 0.000016) and day 90 (*p* = 0.001415) compared at baseline, suggesting the increased *Lactobacillus* species colonization.	Vladareanu et al.^140^
Oral	Total 48 women diagnosed with recurrent BV	All patients were treated with either probiotics (*L. acidophilus* GLA-14 and *L. rhamnosus* HN001) plus lactoferrin or placebo as adjunct to metronidazole for 7 days.	Vaginal tract	6-months cure rate based on Nugent scores: Probiotic 83.3% vs. Placebo 20.8%, *p* < 0.001. 6-months overall cure rate without any symptoms: Probiotic 83.33% vs. Placebo 37.50%, *p* < 0.01. 6-months recurrent rate: Probiotic 29.17% vs. Placebo 58.33%, *p* < 0.05.	Russo et al.^141^
Oral	Total 172 women recently cured of recurrent BV	All patients were treated with either probiotics (*L. crispatus* LMG S-29995, *L. brevis*, and *L. acidophilus*) plus lactoferrin or placebo BID for first 7 days, and one times daily for next 8 to 120 days.	Vaginal tract	Recurrence rate: Probiotic 18.3% vs. Placebo 32.1%, *p* = 0.014. Time to recurrence: Probiotic 97.3 ± 26.7 days vs. Placebo 74.7 ± 27.7 days.	Reznichenko et al.^109^

*BID* twice daily, *BV* bacterial vaginosis, *HPV* Human papillomavirus, *CFU* colony forming unit, *VVC* vulvovaginal candidiasis.

## Evaluating the efficacy of current probiotic therapy in *Helicobacter pylori* infection and dysbacteriosis

### Oral probiotic administration for *Helicobacter pylori* infection and gastrointestinal dysbiosis

Probiotics, defined as “living microorganisms beneficial to the host’s body health,” have emerged as key players in combating foreign pathogens^110^. In recent decades, probiotic therapy has gained recognition for *H. pylori* infection as an effective strategy to enhance eradication rates, mitigate antibiotic-related adverse effects, and lower recurrence rates by restoring normal microbiota in the gastrointestinal tract.

*H. pylori* typically compromise and invade the gastric mucosa, disrupting the mucosal barrier^111^. Certain probiotic strains can stimulate IgA secretion in goblet cells, aiding in mucosal formation and defense against invading pathogens^112^. Strains like *L. plantarum* 299 v and *L. rhamnosus* GG have been shown to enhance the expression of mucin genes MUC2 and MUC3 in gastric epithelial cells^113^, reinforcing the gastrointestinal mucosal barrier. Additionally, probiotics such as *L. acidophilus* NCFM, *L. acidophilus* La-14, *L. plantarum* Lp-115, and *L. rhamnosus* GG exhibit anti-adhesion properties that inhibit urease activity, impeding *H. pylori* colonization^114^. Furthermore, probiotics modulate the inflammatory response triggered by *H. pylori* infection. Strains like *L. crispatus* RIGLD-1^115^ and *L. gasseri* MN-LG80^116^ were reported can alleviate *H. pylori*-induced gastritis by reducing cytokine levels of TNF-α, IL-1β, and IL-6. Probiotics can also aggregate free-moving pathogens, enhancing hindrance to adhesion and increasing susceptibility to phagocytosis. Probiotic strains of *L. rhamnosus* SD11 and *L. paracasei* CNCM I-1572 can effectively co-aggregate and exhibit anti-adhesive properties against *H. pylori* strains^117^. Moreover, *L. salivarius* LN12, when combined with antibiotics, demonstrated the capacity to disrupts biofilm formation in *H. pylori*^118^.

In clinical trials, patients administered with *L. reuteri* DSM 17648 showed significantly higher eradication rates compared to placebo (93.2% vs. 68.9%) for *H. pylori* infection, with reduced side effects^107^. Another study involving bismuth-containing quadruple therapy supplemented with probiotic combinations of *L. reuteri* DSM17938 and *L. reuteri* ATCC PTA6475 reported 96% eradication in the probiotic group compared to 88% in the placebo group following a 14-day therapeutic course^119^. A meta-analysis comprising 9004 patients across 34 trials evaluated the efficacy of antibiotic triple therapy with probiotic supplementation for *H. pylori* eradication^120^. It revealed that combinations of *Lactobacillus* species with triple therapy yielded higher eradication rate compared to triple therapy alone, with *Bifidobacterium*-*Lactobacillus* and *Bifidobacterium*-*Lactobacillus*-*Saccharomyces* combinations achieving eradication rates of 78.3% and 88.2%, respectively. Therefore, the aforementioned results indicate that supplementing conventional therapy with probiotics could significantly enhance *H. pylori* eradication rates and mitigate antibiotic adverse effects.

As both *H. pylori* infection and its eradication therapy can lead to gastrointestinal dysbiosis, resulting in recurrence and susceptibility to opportunistic pathogens, probiotics have demonstrated efficacy in restoring normal microbiota when administered alongside antibiotics^121^. One study conducted by Zhou et al. suggested that administration of *L. paracasei* ZFM 54 significantly reversed *H. pylori*-associated dysbiosis by restoring the abundance of Firmicutes and Actinobacteriota, while decreasing the abundance of Campylobacterota and Proteobacteria^122^. Another multi-centered study revealed that the profound fluctuations of gastric microbiota post bismuth-containing quadruple therapy were significantly mitigated with *Bifidobacterium* Tetravaccine Tablets (contain *B. infantis* CGMCC0460.1, *L. acidophilus* CGMCC0460.2, *Enterococcus faecalis* CGMCC0460.3, and *Bacillus cereus* CGMCC0460.4) supplementation, accompanied by the flourishment of *Lactobacillus*, *Prevotella*, and *Bifidobacterium*^123^. Additionally, another clinical trial indicated significantly enriched microbial diversity in the gastrointestinal tract with *L. rhamnosus* LGG-18 and *L. salivarius* Chen-08 treatment compared to the *H. pylori*-infected group, while gastric proinflammatory responses and premalignant lesions were also profoundly alleviated^124^.

### Intra-vaginal probiotic administration for vaginal dysbacteriosis

Vaginal dysbiosis is characterized by the replacement of dominant *Lactobacillus* microorganisms with obligate or facultative anaerobes like *G. vaginalis* or other pathogenic bacteria^125^. Conventional antibiotic therapies for vaginal dysbiosis are insufficient and can exacerbate dysbiosis. Hence, alternative bioactive preparations, such as probiotics alone or in combination with antibiotics, are emerging as viable strategies for addressing vaginal dysbiosis.

**Fig. 3:**
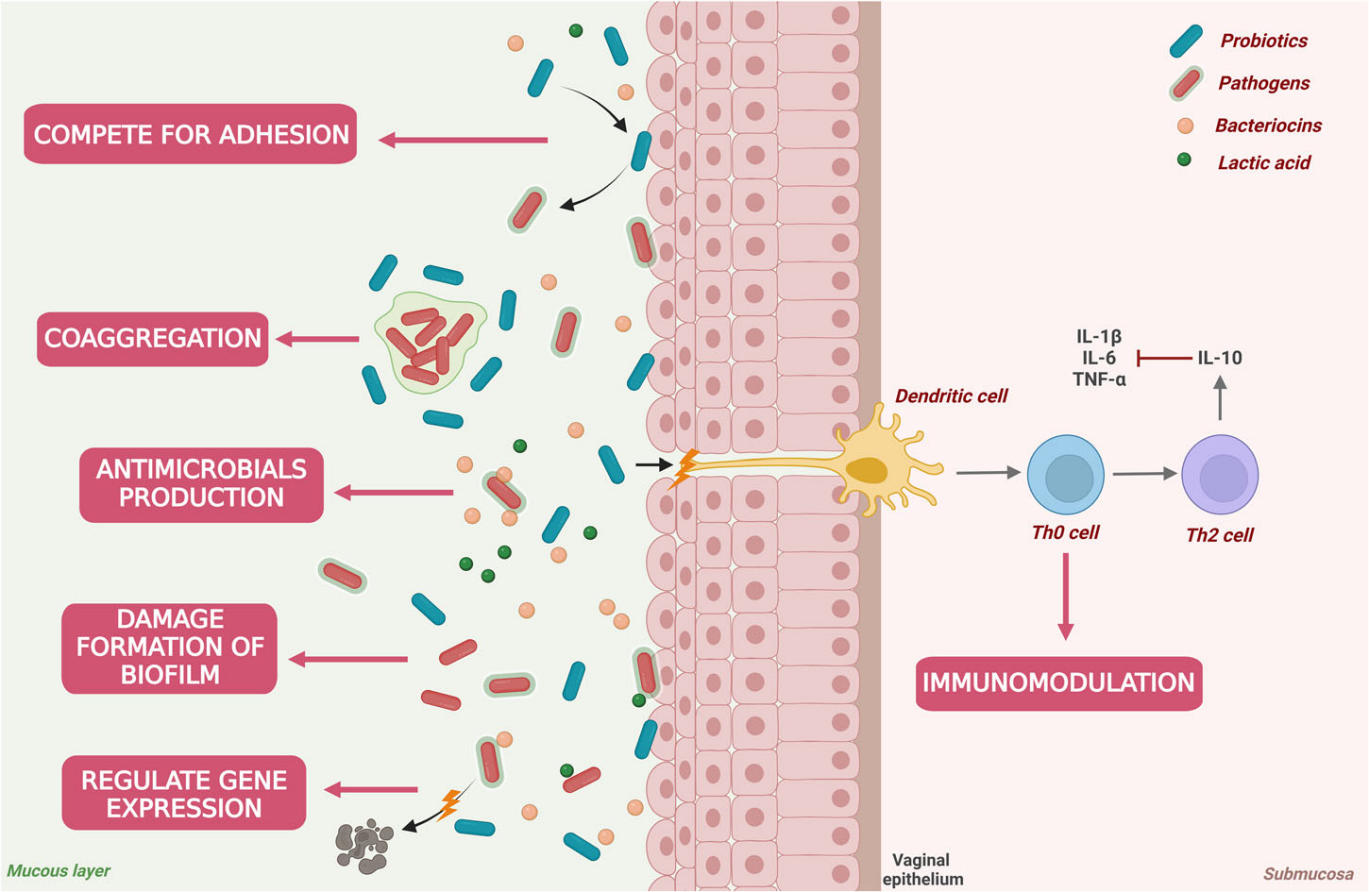
Mechanisms of probiotics regulate vaginal microbiota. Vaginal probiotics can operate through adhesive competition, coaggregation, antimicrobial production, direct disruption of bacterial biofilm formation, regulation of bacterial gene expression, and immunomodulation to regulate the vaginal microenvironment.

Similar as oral probiotics targeting *H. pylori*, intra-vaginally administered probiotics demonstrate efficacy against vaginal dysbiosis through mechanisms such as coaggregation with pathogens, immunomodulation, antimicrobial production, disruption of pathogenic biofilm formation, and gene expression modulation (Fig. 3). For instance, studies have shown that certain *Lactobacillus* strains (*L. delbrueckii* ATCC14917, *L. plantarum* DM8909, and *L. plantarum* ZX27) can significantly inhibit the growth of *G. vaginalis* through coaggregation, contributing to the restoration of vaginal ecological balance^126^. Additionally, probiotics like *L. rhamnosus* IDCC 3201 exhibit immunomodulatory effects and can inhibit vaginal pathogens like *C. albicans*^127^. Besides immunomodulatory effects and co-aggregation, probiotics can maintain normal vaginal ecology by producing diverse antimicrobials such as H_2_O_2_ and bacteriocins^128^. Further, certain reports have indicated that strains like *L. kefiranofaciens* DD2131^129^ and *L. helveticus* HY7801^130^ can hinder the normal metabolism of *G. vaginalis* and disrupt biofilm formation. At the genetic level, a study revealed that treatment with *L. crispatus* EX533959VC06 downregulated the expression of vaginolysin (*vly*) in *G. vaginalis*, leading to a significant reduction in its cytotoxicity and adhesive properties in the vaginal environment, thereby mitigating the risk of bacterial vaginosis^131^.

Additionally, emerging evidence suggests that intra-vaginal probiotic administration can serve as an adjunct or alternative to antibiotic therapy for treating vaginal dysbacteriosis. A meta-analysis conducted by Jeng and colleagues revealed a significantly higher cure rate within one month of treatment among individuals supplemented with probiotics (OR = 4.55, 95% CI: 1.44–14.36, *p* = 0.010)^132^. Another meta-analysis comprising 12 trials also indicated a promising potential of vaginal probiotics in treating bacterial vaginosis^133^. A randomized controlled trial by Sgibnev and colleagues demonstrated that combining vaginal-administered *L. rhamnosus* Lcr35 with antimicrobial therapy significantly improve the cure rate of *Trichomonas vaginalis* (88.6% vs. 42.9%) and bacterial vaginosis (63.6% vs. 11.9%)^108^. Subsequent investigations suggested that probiotic supplementation could further restore the vagina’s physicochemical parameters to normal levels. Moreover, Bohbot et al. reported a significantly lower recurrence rate in the *L. crispatus* IP174178 group (20.5%) compared to the placebo group (41%)^134^. Additionally, the time to recurrence was significantly longer in the probiotic group (3.75 ± 0.16 months) relative to the placebo group (2.93 ± 0.18 months, *p* = 0.0298). Palma et al. also suggested that the long-term intra-vaginally application of *L. rhamnosus* BMX 54 could retore the vaginal microbial eubiosis^135^.

### Oral probiotic administration for vaginal dysbacteriosis

In addition to vaginal administration, oral consumption of probiotics is more practical, as it is more user-friendly and can also be an effective approach to maintain vaginal eubiosis^136^. Ho et al. evaluated the daily oral administration of probiotic combinations (*L. rhamnosus* GR-1 and *L. reuteri* RC-14) in pregnant women to reduce vaginal colonization of Group B *Streptococcus* (GBS). Their findings revealed that 42.9% of patients in the probiotic group exhibited negative GBS colonization in the vagina, compared to 18.0% in the placebo group, suggesting that oral probiotics could diminish pathogen colonization in the vagina^137^. Moreover, oral administration of *L. acidophilus* CBT LA1, *L. rhamnosus* CBT LR5, and *L. reuteri* CBT LU4 significantly improved vaginal dysbiosis in asymptomatic women and restored the abundance of *Lactobacillus* spp., resulting in a healthier vaginal microenvironment post-treatment^138^. Additionally, a study investigating probiotic supplementation for bacterial vaginosis and vulvovaginal candidiasis (VVC) demonstrated similar efficacy. Combinations of *L. crispatus* DSM32720, *L. crispatus* DSM32718, and *L. crispatus* DSM32716 notably alleviated VVC-associated symptoms, reducing discharge and itching. Similarly, combinations of *L. crispatus* DSM32717 and *L. crispatus* DSM32720 reduced episodes of BV, increased vaginal abundance of *Lactobacillus* species, and decreased BV-correlated bacteria^139^. A study by Vladareanu et al. also indicated that oral consumption of *L. plantarum* P17630 restored vaginal colonization of lactic acid-producing bacteria and improved signs of VVC^140^.

Further, oral probiotic therapy has demonstrated greater efficacy in addressing recurrent vaginal dysbiosis. In a randomized study by Russo et al.^141^, the probiotic combination (*L. acidophilus* GLA-14 and *L. rhamnosus* HN001) used as an adjunct to metronidazole showed significantly improved BV-associated symptoms (such as vaginal discharge and itching) and a significantly reduced recurrence rate compared to the placebo group (29.17% vs. 58.33%) during the 6-month follow-up period. Additionally, another study found that the overall rate of recurrent episodes was 18.3% in the probiotic group (receiving oral administration of *L. crispatus* LMG S-29995, *L. brevis*, and *L. acidophilus*), whereas it was 32.1% in the placebo group. The mean time to BV recurrence was 97.3 days in the probiotic group and 74.7 days in the placebo group, indicating that oral probiotic supplementation was associated with a prolonged interval between recurrences and a reduced recurrence rate^109^.

Research investigating the underlying mechanism by which oral probiotics affect the vaginal microenvironment is inadequate. One assumption is that orally administered probiotics directly translocate from the rectum to the vagina and colonize it. In a study by Strus et al.^99^, molecular methods were employed to evaluate the degree and persistence of colonization of a probiotic mixture consisting of *L. fermentum* 57 A, *L. plantarum* 57B, and *L. gasseri* 57 C. They found that with improved vaginal physiological parameters, the first detection of at least one applied strain colonizing the vaginal epithelium occurred at day 10 (in 2 out of 25 participants) since the start of probiotic administration. The number of colonization peaked at day 31 (in 15 out of 25 participants), and colonization persisted until day 70 (in 5 out of 25 participants). This suggests that probiotics could pass through the gastrointestinal tract, adhere to the vaginal epithelium for weeks, and be associated with the improvement of vaginal microbiota. However, studies by Yefet et al.^142^ and Koirala et al.^143^ reported relatively low signs of vaginal colonization of oral probiotics in their volunteers, suggesting that the mechanism of direct translocation might not be applicable for all probiotic strains. Another study that orally administered *L. gasseri* TM13 and *L. crispatus* LG55 as adjuncts to metronidazole indicated that the probiotic group demonstrated profound restoration of vaginal health. Although there was a significant enrichment of intestinal microbiota, the probiotics were not identified within the vaginal microbiota, suggesting that the therapeutic effect of *L. gasseri* TM13 and *L. crispatus* LG55 may act through the gastrointestinal microbiota^144^. This finding might correlate with the previously mentioned indirect association between the vaginal and gut microbiota *via* the vagina-gut axis.

### Exploring the potential of intra-vaginal probiotic administration for gastrointestinal dysbiosis

As of now, there is limited solid evidence to conclusively demonstrate that intra-vaginal probiotic administration could directly affect the gastrointestinal tract. Most research on probiotics focuses on their impact on the local microbiota in the area where they are administered. Some studies have suggested potential indirect effects or systemic interactions between the vaginal and gastrointestinal microbiota. Through vagina-gut axis, alterations in the vaginal microbiota might influence systemic immune responses or microbial translocation, which could in turn affect the gastrointestinal microbiota.

Ang et al. reported that female patients with vaginal candidiasis exhibited not only a compromised vaginal microbial community but also a significantly altered gut microbial profile with reduced microbial diversity, indicating that perturbations in vaginal microecology could, in turn, affect gut microecology^36^. Further, another study^102^ implicated *G. vaginalis* infection in mice vagina increased the inflammatory profile in colon tissue with elevated TNF-α and myeloperoxidase activity, and reduced IL-10. Additionally, this infection also led to a decrease in the abundance of Bacteroidetes and an increase in the abundance of Proteobacteria in the gastrointestinal tract. Moreover, since IgA coating is crucial for microbial colonization in the gut^145^, and IL-5 is associated with the vaginal abundance of *Prevotella* spp^146^., which is involved in IgA responses^147^, the disturbance of vaginal microbiota may accordingly affect gastrointestinal microbiota through systemic immune responses. Numerous studies have also demonstrated that vaginal probiotic delivery can induce systemic anti-inflammatory effects, which may benefit conditions such as endometriosis, cervical cancer, and overactive bladder syndrome^148^. Further, given the close proximity between the rectal and vaginal tracts, intra-vaginal probiotics might have the potential to migrate to the gastrointestinal tract through mechanical movement^101^. Considering these factors, it is plausible to speculate that vaginal administration of probiotics could have a beneficial impact on the readjustment of the gastrointestinal microbiota. However, these mechanisms are not yet fully understood, and further research is needed to elucidate the extent of such interactions and their clinical significance.

## Challenges and limitations of dual-channel probiotic therapy

Potential difficulties and drawbacks of dual-channel probiotic therapy need to be carefully considered despite its promising prospects. For instance, the incidence of vaginal dysbiosis outbreaks is relatively low compared to the total number of female patients treated with antibiotics, indicating that patients may prefer conventional therapy over dual-channel probiotic therapy. Another significant challenge lies in the complexity of coordinating both oral and intra-vaginal administration routes, which may lead to issues such as inconsistent dosing regimens and patient compliance. Furthermore, the cost and accessibility of probiotic supplements may present barriers to widespread adoption, particularly in resource-limited area. The most critical issue is the lack of validated efficacy and safety of current probiotic therapy due to our inadequate understanding of the mechanism of action of probiotics.

The effectiveness of probiotics may vary depending on individual factors such as gut and vaginal microbiota composition, underlying health conditions, and lifestyle factors, posing a challenge in achieving consistent therapeutic outcomes. Different strains of probiotics also exhibit varying efficiencies in eradicating pathogens in specific individuals, making it difficult for doctors to devise a tailored regimen. Moreover, probiotics need to colonize the mucosal layer of the local tract so that they can persistently function to achieve favorable clinical results. However, for dual-channel probiotic therapy, regardless of the chance that oral and intra-vaginal dosed probiotics can colonize the gastrointestinal and vaginal tracts, there is no sufficient evidence to support that oral probiotics can eventually reside in the vaginal tract, and no report suggests that a vaginal probiotic can colonize into gastrointestinal tract. Further, some studies have presented conflicting viewpoints on the actual efficacy of probiotics in improving *H. pylori* eradication and vaginal dysbiosis^149^. For example, a meta-analysis involving 2491 papers suggested that probiotics in standard triple therapy for *H. pylori* infection did not assist in the eradication of *H. pylori* compared with the placebo group (*p* = 0.816)^150^.

Additionally, there is limited research investigating the long-term safety and potential adverse effects of concurrent oral and intra-vaginal probiotic administration. Despite probiotics being generally regarded as safe, there is a possibility of adverse effects, particularly when administered in high doses or in individuals with compromised immune systems. Concurrent oral and intra-vaginal administration may increase the risk of adverse reactions, such as probiotic infection^151,152^, gastrointestinal discomfort^153,154^, allergic reactions^155^, or dysbiosis, which need to be carefully monitored. Many different clinical risks are related to probiotic supplementation. Specifically, *Lactobacillus* GG, *L. acidophilus*, *L. casei* are the most reported strains that can lead to bacteriaemia^156–158^. A meta-analysis containing 60 clinical cases and a total of 93 patients discovered that *Lactobacillus* and *Bifidobacterium* are the second and third main agents for bacteremia, respectively, with 26 (27.9%) and 12 (12.8%) in total involved cases^159^. Another major risk factor is gene transfer between dosed probiotics and commensal bacteria in the gastrointestinal tract of the host, which can result in the acquisition of drug resistance by pathogens. Antibiotic resistance genes, such as *erm* and *tet* which belong to *Lactobacillus* and *Bifidobacterium* genera, have been found to exist in commensal pathogens in the gut microbiota^160,161^. With dual-channel delivery, there is a greater chance of probiotic-related adverse effects. Therefore, the safety profile of dual-channel therapy requires further investigation.

Furthermore, incorporating intra-vaginal probiotic administration into treatment regimens may raise concerns or discomfort among patients, impacting their acceptance and adherence to therapy. Education, counseling, and clear communication are essential to address patient preferences and ensure optimal compliance with dual-channel probiotic therapy. Dual-channel probiotic therapy also raises ethical considerations regarding patient autonomy, informed consent, and equitable access to care. Clinicians must ensure that patients are fully informed about the benefits, risks, and alternatives of this treatment approach throughout the decision-making process. Overall, while dual-channel probiotic therapy holds promise, addressing these challenges is essential to maximize its potential benefits in clinical practice.

In summary, *H. pylori* infection poses significant risks to gastric health, while the dysbiosis resulting from *H. pylori* eradication therapy can also negatively impact vaginal health. Conventional antibiotic treatments for these conditions have shown limited efficacy and often fail to provide lasting or comprehensive remission. Given the interconnectedness of the vaginal and gastrointestinal microbiota *via* the vagina-gut axis, as well as the effectiveness of oral probiotics in addressing both *H. pylori* infection and vaginal dysbiosis, and the potential of intra-vaginal probiotics to treat vaginal dysbiosis and possibly gastrointestinal dysbiosis, simultaneous oral and vaginal probiotic therapy may emerges as a promising approach. This dual-channel probiotic therapy holds the promise of enhancing the eradication rate of *H. pylori* infection while decreasing the likelihood of gastrointestinal and vaginal dysbiosis outbreaks. However, several challenges and limitations must be addressed before widespread adoption can be realized. Continued research efforts are warranted to fully understand its clinical utility and optimize its implementation in clinical practice. With further refinement and validation, dual-channel probiotic therapy may ultimately offer a safe, effective, and holistic approach to managing microbial dysbiosis and improving patient outcomes in both gastrointestinal and vaginal health.
